# Supplementary material for: General negative pressure annealing approach for creating ultra-high-loading single atom catalyst libraries
Source: Nat Commun. 2024 Jul 6;15:5675. doi: 10.1038/s41467-024-50061-1 (PMC11227521; doi:10.1038/s41467-024-50061-1)
Supplement: Supplementary file 1 — Supplementary Information [file 41467_2024_50061_MOESM1_ESM.pdf]

Supporting information

General Negative Pressure Annealing Approach for Creating Ultra-high-loading Single Atom Catalyst Libraries

Yi Wang[1]†, Chongao Li[1]†, Xiao Han[2]†, Jintao Bai[1], Xuejing Wang[3], Lirong Zheng[4], Chunxia Hong[5], Zhijun Li[6], Jinbo Bai[7], Kunyue Leng[1]*, Yue Lin[2]*, Yunteng Qu[1]*

[1]*International Collaborative Center on Photoelectric Technology and Nano Functional Materials, Institute of Photonics and Photon-Technology, Northwest University, Xi'an, Shaanxi 710069, China*

[2]*Hefei National Research Center for Physical Sciences at the Microscale, Department of Chemistry, Department of Applied Chemistry, University of Science and Technology of China, Hefei, Anhui 230026, P.R. China*

[3]*Interdisciplinary Research Center of Biology & Catalysis, School of Life Sciences, Northwestern Polytechnical University, Xi'an 710000, China*

[4]*Institute of High Energy Physics, Beijing 100039, China*

[5]*Shanghai Advanced Research Institute, Chinese Academy of Science, Shanghai 201210, China*

[6]National Key Laboratory of Continental Shale Oil, College of Chemistry and Chemical Engineering, Northeast Petroleum University, Daqing 163318, P. R. China

[7]*Université Paris-Saclay, CentraleSupélec, ENS Paris-Saclay, CNRS, LMPS-Laboratoire de Mécanique Paris-Saclay, 8-10 rue Joliot-Curie, Gif-sur-Yvette 91190, France*

Corresponding Authors: Kunyue Leng (lengky@nwu.edu.cn), Yue Lin (linyue@ustc.edu.cn), Yunteng Qu (yuntengqu@nwu.edu.cn)

† These authors contribute to this work equally.

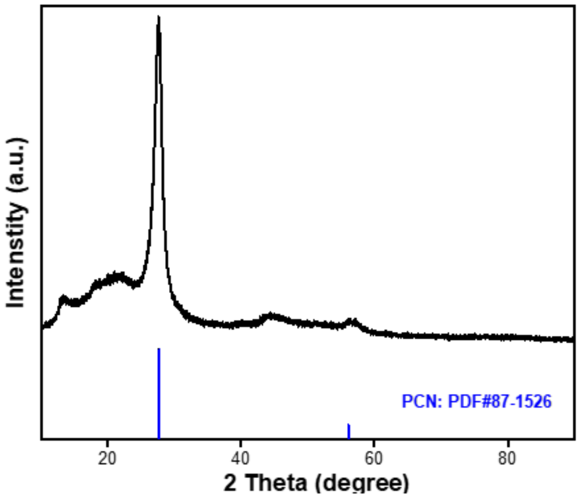

Supplementary Figure 1. XRD pattern of the polymeric carbon nitride substrate. The peaks at 2θ of 27.6 and 60.0 degrees belong to the characteristic diffraction of PCN.

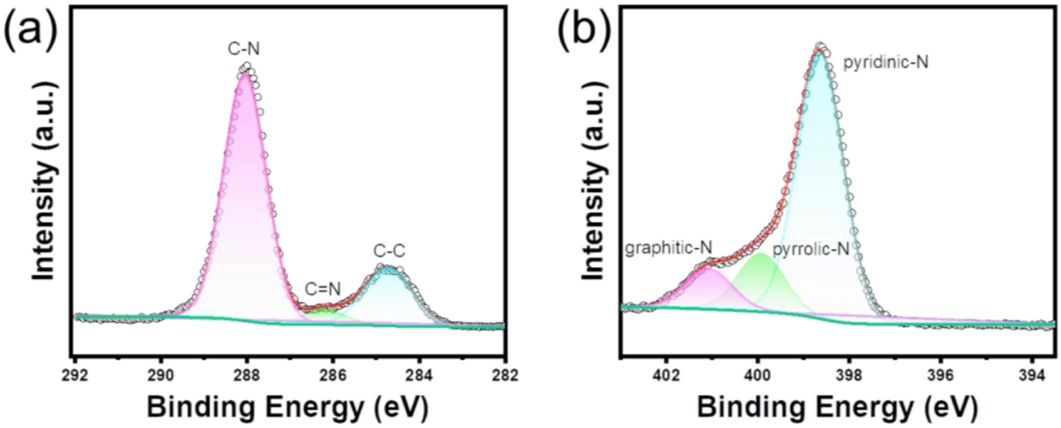

Supplementary Figure 2. (a) C 1*s*, (b) N1*s* XPS spectra of the polymeric carbon nitride. The C-N coordination is detected.

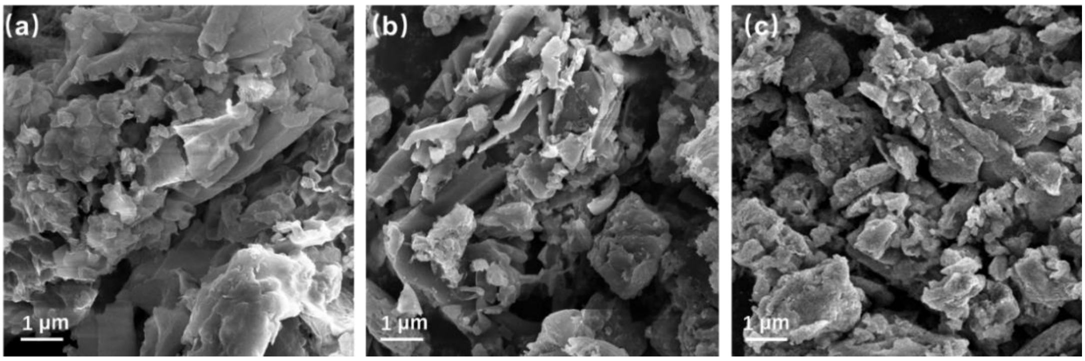

Supplementary Figure 3. The SEM images of (a) Pt SACs/PCN, (b) Pt NPs/PCN and (c) PCN. All three samples show similar apparent morphology.

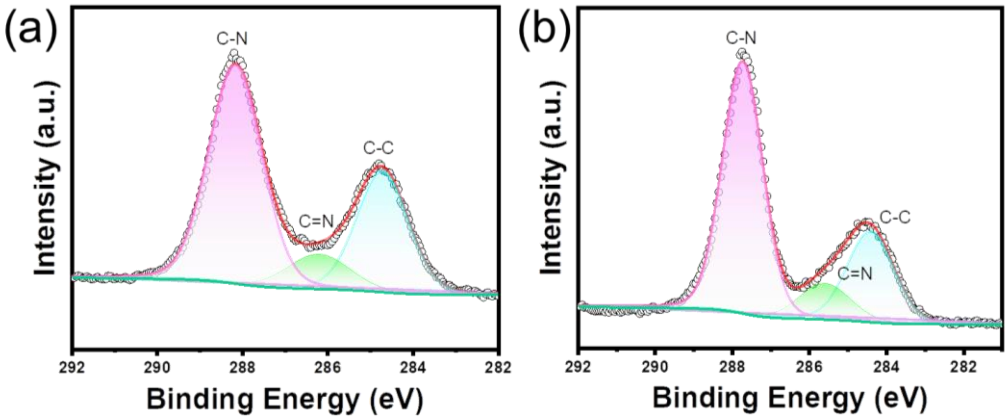

Supplementary Figure 4. (a) C 1*s* spectra of (a) Pt SACs/PCN, (b) Pt NPs/PCN. The annealing condition show negligible impaction on the C 1*s* spectra.

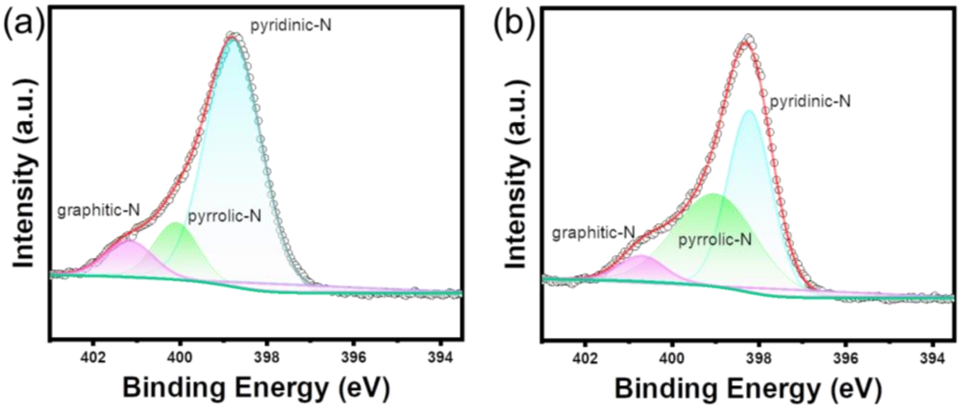

Supplementary Figure 5. N1*s* XPS spectra of (a) Pt SACs/PCN, (b) Pt NPs/PCN. The annealing condition show negligible impaction on the N 1*s* spectra.

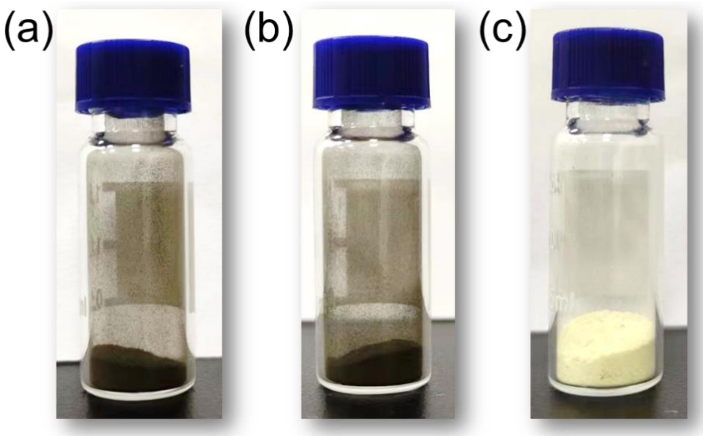

Supplementary Figure 6. The digital photograph of (a) Pt SACs/PCN, (b) Pt NPs/PCN and (c) PCN. After Pt loading the colour turns black compare with original PCN.

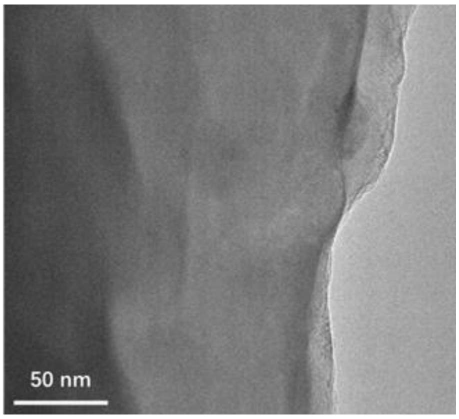

Supplementary Figure 7. TEM images of Pt SACs/PCN, no metal particles are detected.

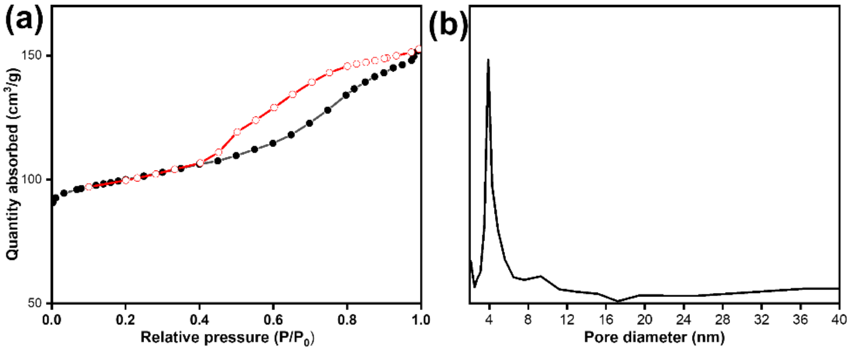

Supplementary Figure 8. (a) N_2 adsorption/desorption isotherms and (b) pore size distirbution of PCN. The BET surface area of PCN is used to edtimate the areal density of isolated metal sites in M SACs/PCN catalysts.

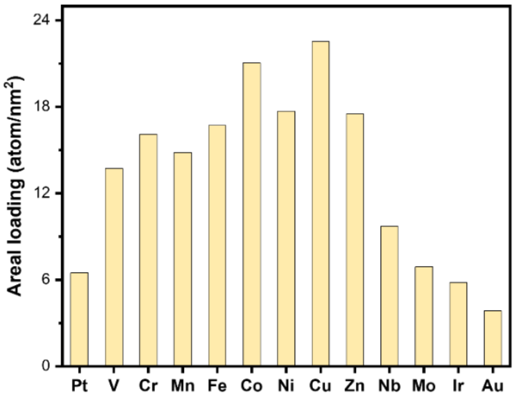

Supplementary Figure 9. Areal density of metal atoms in UHL-SACs based on PCN carrier. The Areal density was estimated based on the bulk metal content and the specific surface area of the carrier, assuming that all of the metal remains on the carrier surface. Although wet deposition approaches are known to promote surface localization, the metals were predicted to percolate preferentially into the bulk of graphitic carbon nitride (*ACS Catal.* 2020, **10**, 11069, and *Nat. Nanotech.*, 2022, **17**, 174-181.), which might explain the relative high value.

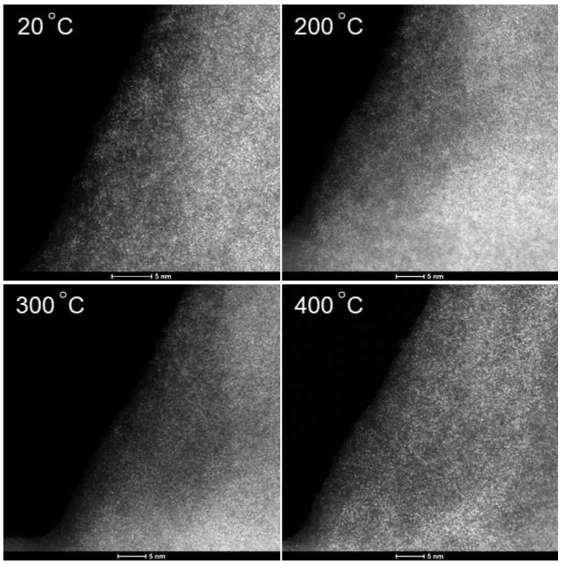

Supplementary Figure 10. Temperature-dependent in-situ aberration-corrected HAADF-TEM images of Pt SACs/PCN. No clusters and particles are generated along the temperature increasing from 20 to 400 °C

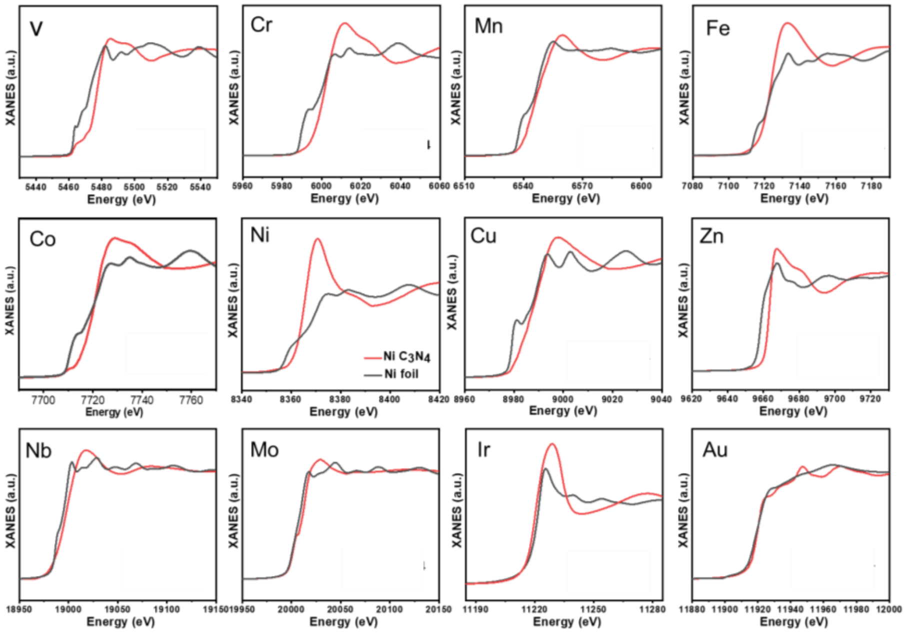

Supplementary Figure 11. XAENs spectra of M SACs/PCN, M=V, Cr, Mn, Fe, Co, Ni, Cu, Zn, Nb, Mo, Ir and Au. Red line represents the as prepared catalyst and black line represents metal foil. All samples show positive oxidation state based on the comparison on the intensity of the white line.

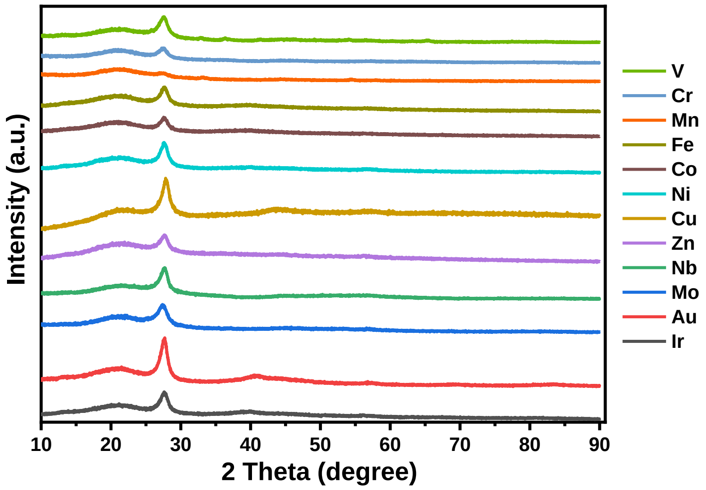

Supplementary Figure 12. XRD pattern of of M SACs/PCN, M=V, Cr, Mn, Fe, Co, Ni, Cu, Zn, Nb, Mo, Ir and Au. All sample show only characteristic diffraction peaks of PCN.

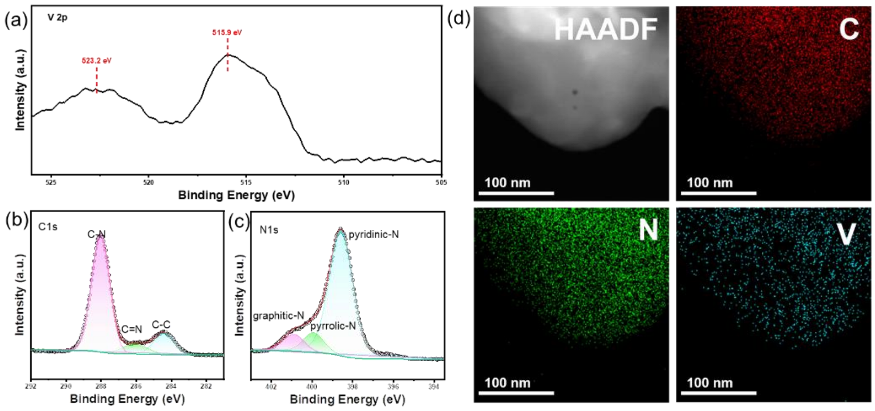

Supplementary Figure 13. Characterizations of V SACs/PCN. (a-c) XPS spectra. (d) HAADF-TEM and EDS element mapping. The metal species distribute uniformly on the PCN substrate with a positive oxidation state.

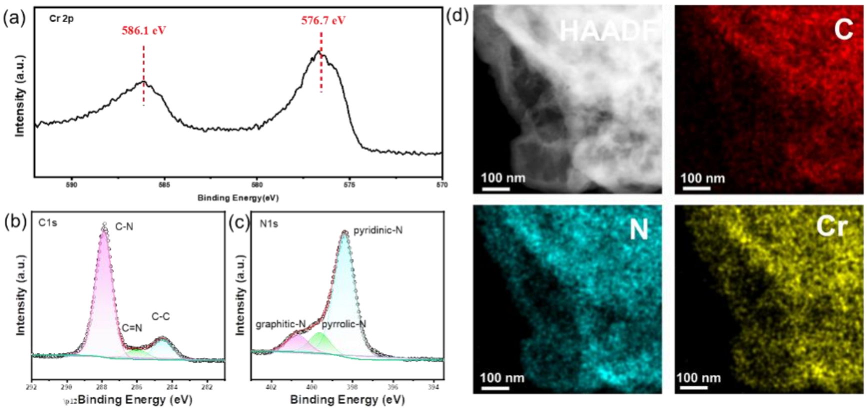

Supplementary Figure 14. Characterizations of Cr SACs/PCN. (a-c) XPS spectra. (d) HAADF-TEM and EDS element mapping. The metal species distribute uniformly on the PCN substrate with a positive oxidation state.

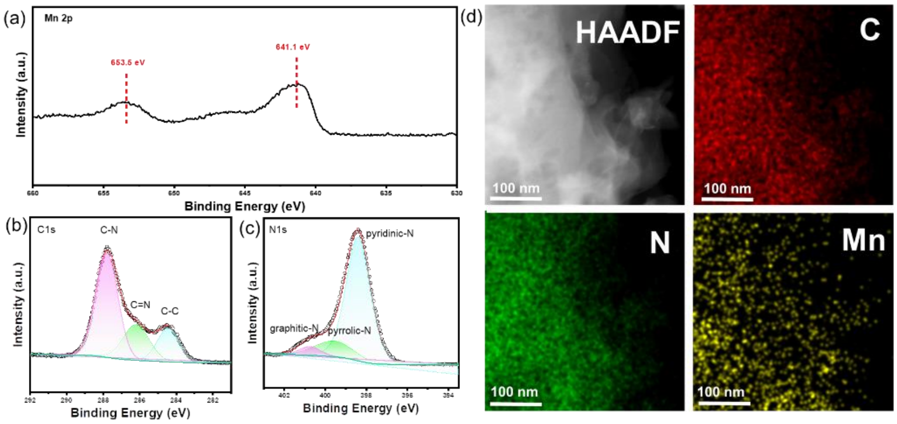

Supplementary Figure 15. Characterizations of Mn SACs/PCN. (a-c) XPS spectra. (d) HAADF-TEM and EDS element mapping. The metal species distribute uniformly on the PCN substrate with a positive oxidation state.

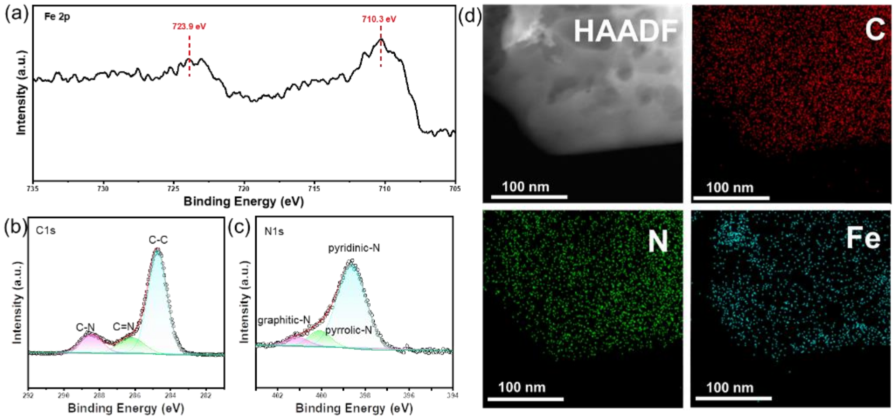

Supplementary Figure 16. Characterizations of Fe SACs/PCN. (a-c) XPS spectra. (d) HAADF-TEM and EDS element mapping. The metal species distribute uniformly on the PCN substrate with a positive oxidation state.

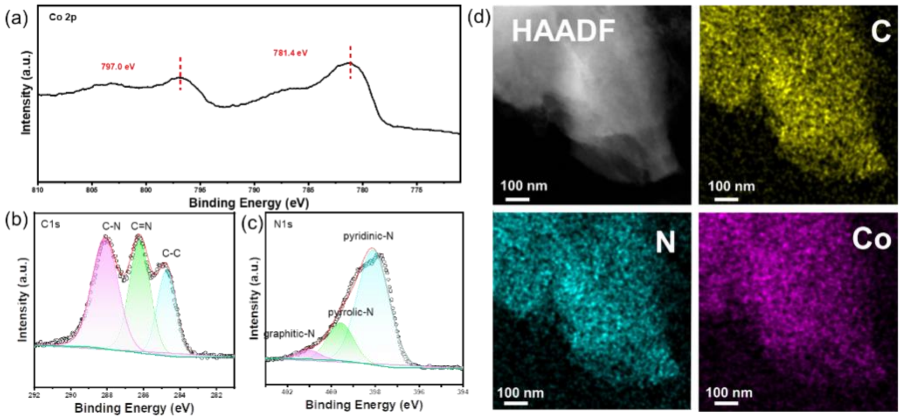

Supplementary Figure 17. Characterizations of Co SACs/PCN. (a-c) XPS spectra. (d) HAADF-TEM and EDS element mapping. The metal species distribute uniformly on the PCN substrate with a positive oxidation state.

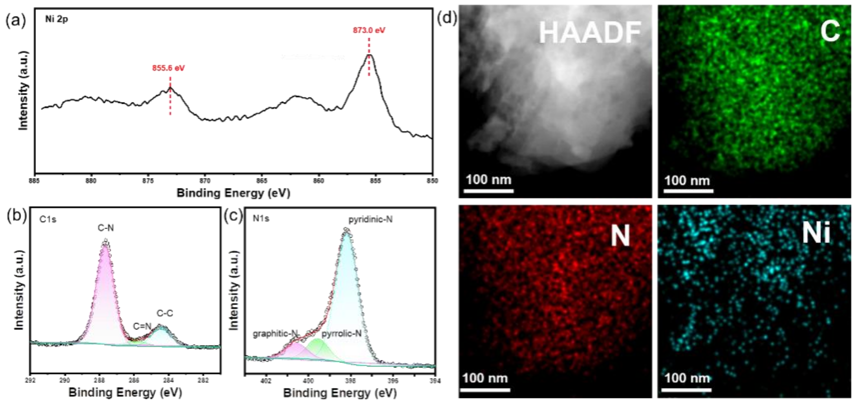

Supplementary Figure 18. Characterizations of Ni SACs/PCN. (a-c) XPS spectra. (d) HAADF-TEM and EDS element mapping. The metal species distribute uniformly on the PCN substrate with a positive oxidation state.

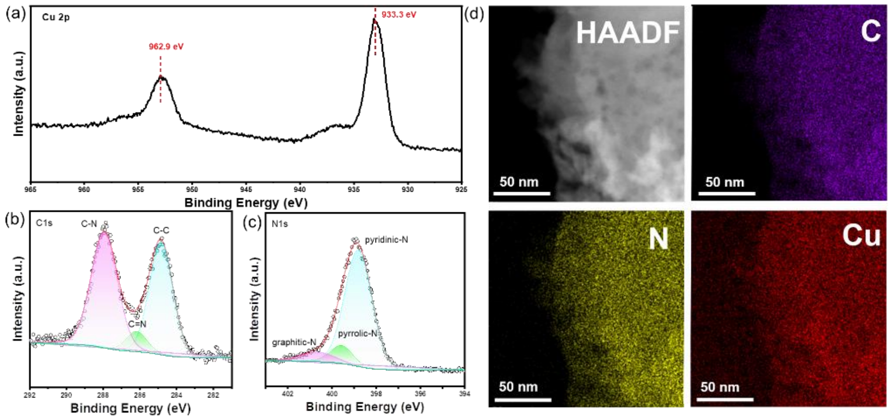

Supplementary Figure 19. Characterizations of Cu SACs/PCN. (a-c) XPS spectra. (d) HAADF-TEM and EDS element mapping. The metal species distribute uniformly on the PCN substrate with a positive oxidation state.

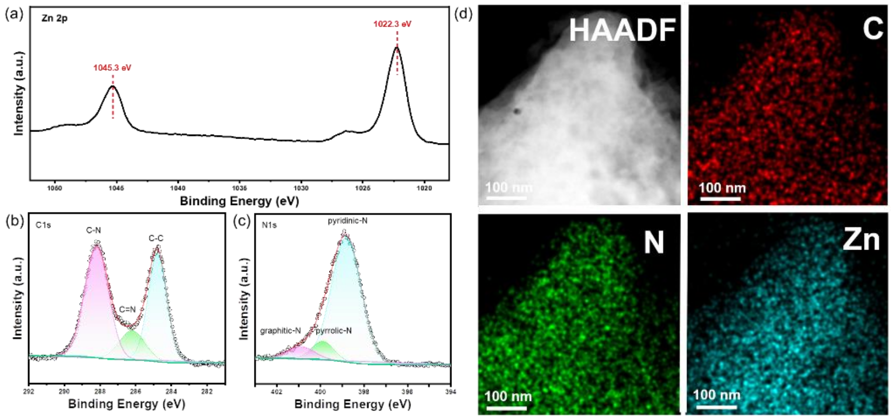

Supplementary Figure 20. Characterizations of Zn SACs/PCN. (a-c) XPS spectra. (d) HAADF-TEM and EDS element mapping. The metal species distribute uniformly on the PCN substrate with a positive oxidation state.

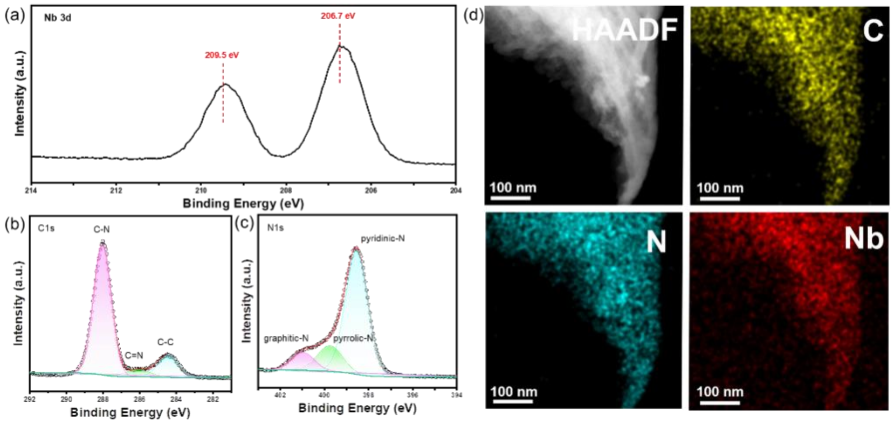

Supplementary Figure 21. Characterizations of Nb SACs/PCN. (a-c) XPS spectra. (d) HAADF-TEM and EDS element mapping. The metal species distribute uniformly on the PCN substrate with a positive oxidation state.

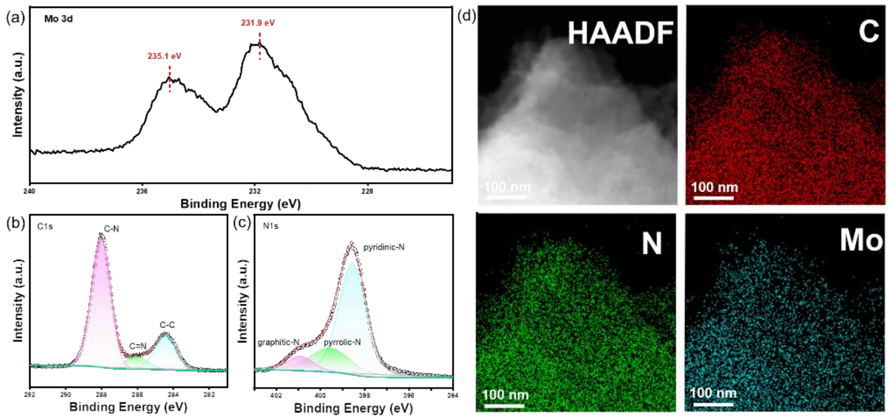

Supplementary Figure 22. Characterizations of Mo SACs/PCN. (a-c) XPS spectra. (d) HAADF-TEM and EDS element mapping. The metal species distribute uniformly on the PCN substrate with a positive oxidation state.

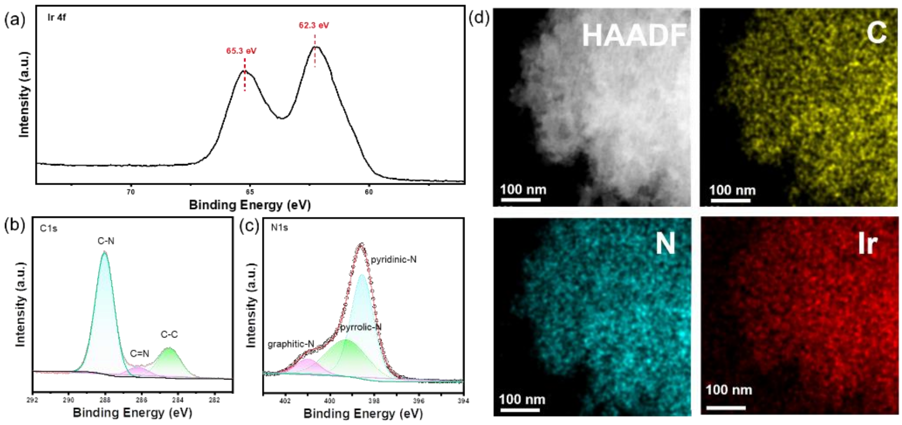

Supplementary Figure 23. Characterizations of Ir SACs/PCN. (a-c) XPS spectra. (d) HAADF-TEM and EDS element mapping. The metal species distribute uniformly on the PCN substrate with a positive oxidation state.

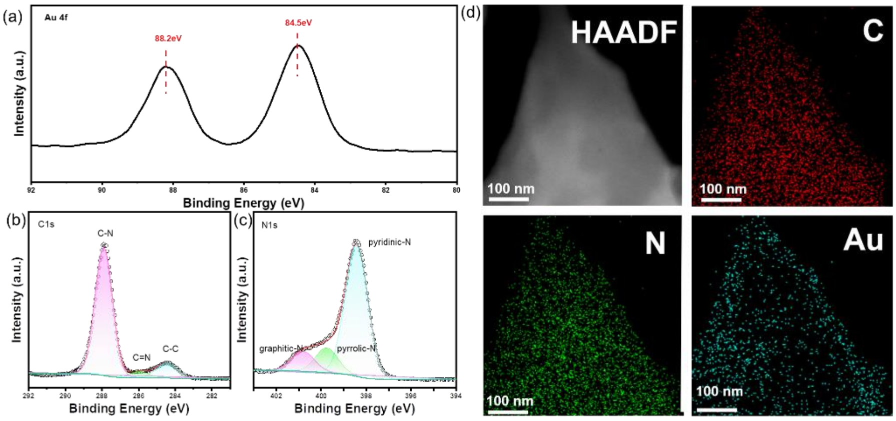

Supplementary Figure 24. Characterizations of Au SACs/PCN. (a-c) XPS spectra. (d) HAADF-TEM and EDS element mapping. The metal species distribute uniformly on the PCN substrate with a positive oxidation state.

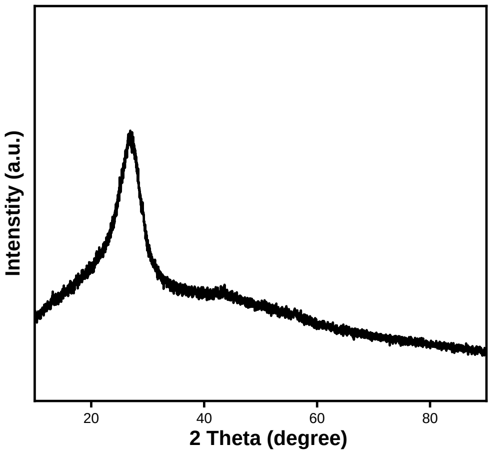

Supplementary Figure 25. XRD pattern of N-dopped carbon (NC), confirming the formation of graphitic carbon.

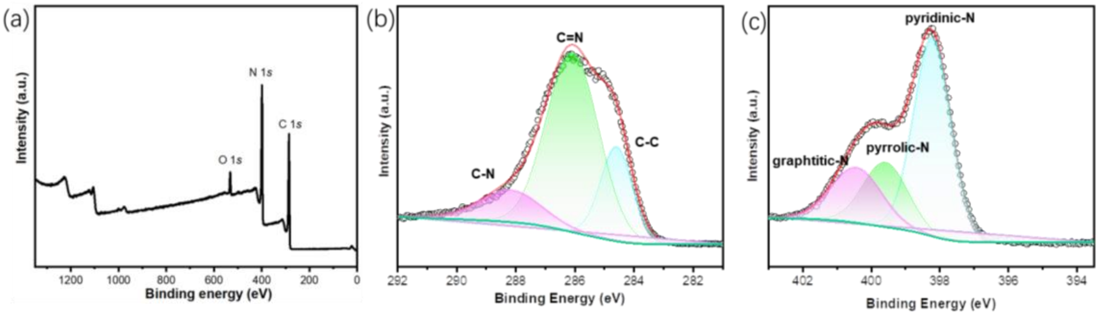

Supplementary Figure 26. XPS results of the N-dopped carbon (NC) substrate. (a) Survey. (b) C 1*s* spectra. (c) N 1*s* spectra. The N-C coordination is indicated, revealing the N doping onto graphitic carbon.

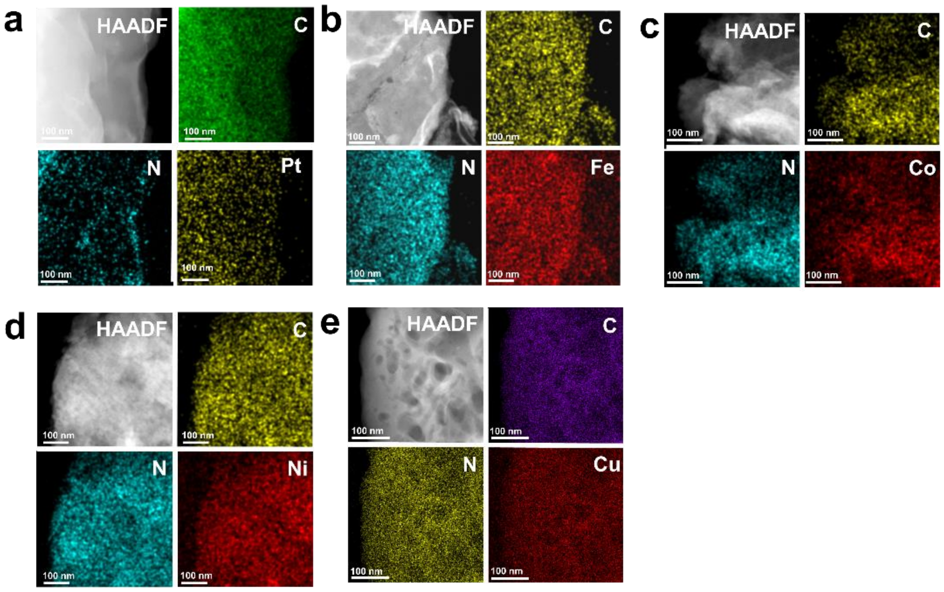

Supplementary Figure 27. EDS element mapping of M SAC/NC, M=Pt, Fe, Co, Ni and Cu. All metals are distributed uniformly on the NC.

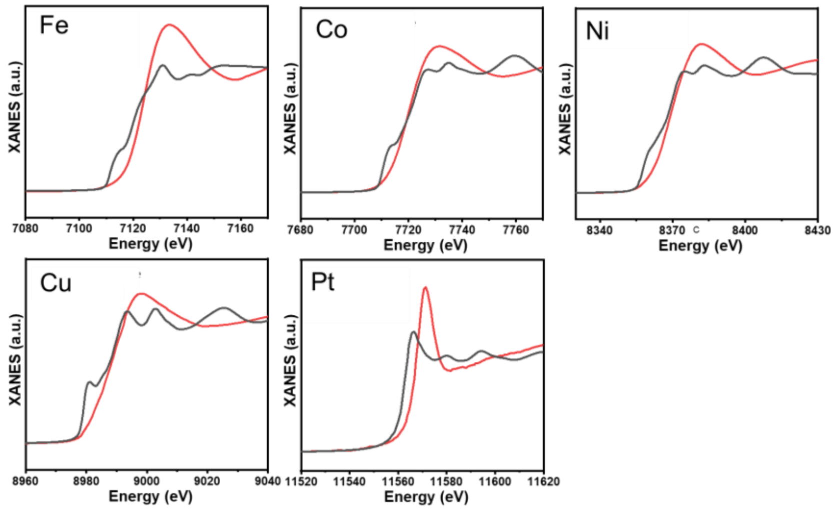

Supplementary Figure 28. XAENs spectra of M SAC/NC, M=Pt, Fe, Co, Ni and Cu. Red line represents the as prepared catalyst and black line represents metal foil. All samples show positive oxidation state based on the comparison on the intensity of the white line.

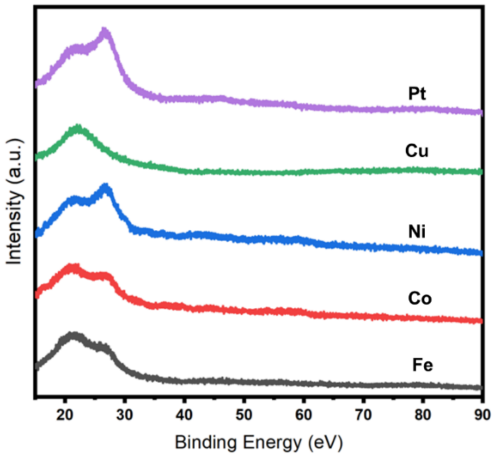

Supplementary Figure 29. XRD pattern of M SAC/NC, M=Pt, Fe, Co, Ni and Cu, no crystalline metal is detected.

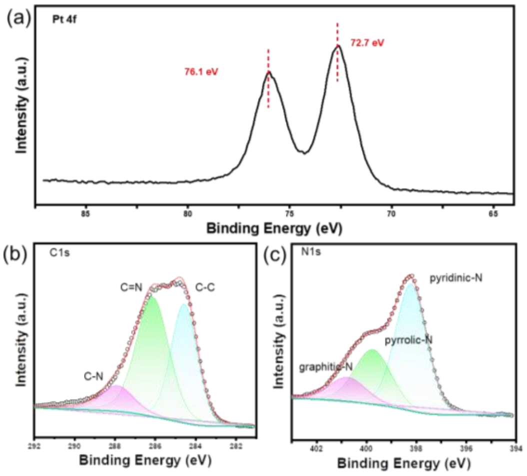

Supplementary Figure 30. XPS spectra of Pt SAC/NC. Metal species with positive oxidation state is confirmed on the N-dopped carbon.

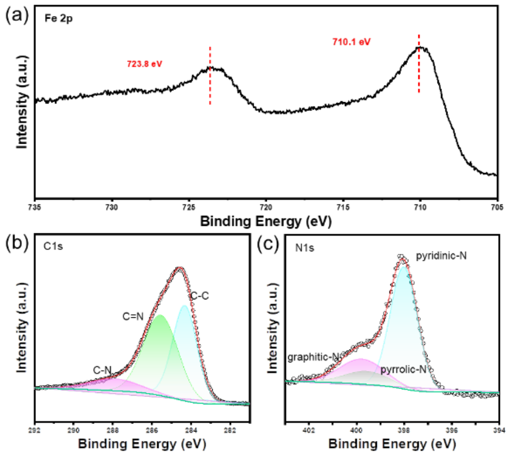

Supplementary Figure 31. XPS spectra of Fe SAC/NC. Metal species with positive oxidation state is confirmed on the N-dopped carbon.

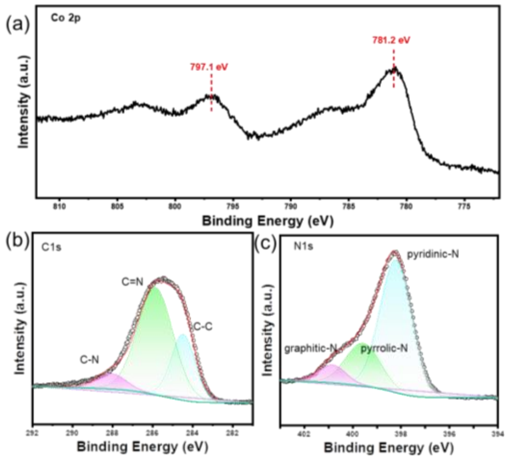

Supplementary Figure 32. XPS spectra of Co SAC/NC. Metal species with positive oxidation state is confirmed on the N-dopped carbon.

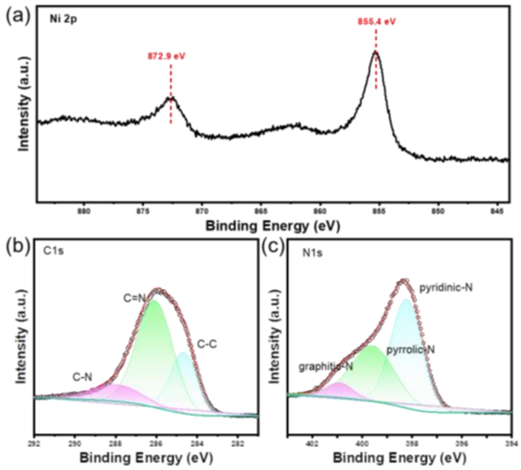

Supplementary Figure 33. XPS spectra of Ni SAC/NC. Metal species with positive oxidation state is confirmed on the N-dopped carbon.

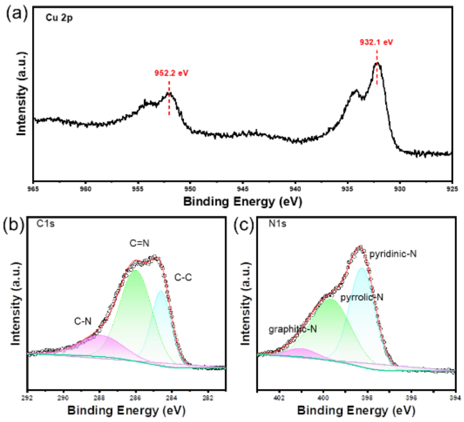

Supplementary Figure 34. XPS spectra of Cu SAC/NC. Metal species with positive oxidation state is confirmed on the N-dopped carbon.

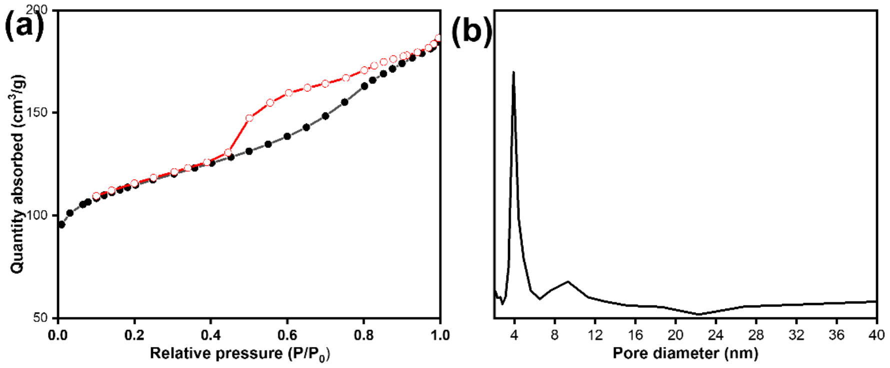

Supplementary Figure 35. (a) N_2 adsorption/desorption isotherms and (b) pore size distirbution of NC. The BET surface area of NC is used to edtimate the areal density of isolated metal sites in M SACs/NC catalysts.

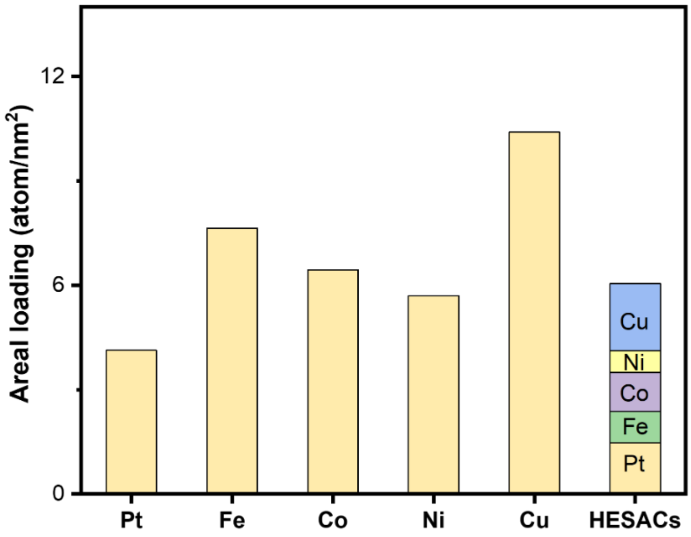

Supplementary Figure 36. Areal density of metal atoms in UHL-SACs based on NC carrier. The Areal density was estimated based on the bulk metal content and the specific surface area of the carrier, assuming that all of the metal remains on the carrier surface. Although wet deposition approaches are known to promote surface localization, the metals were predicted to percolate preferentially into the bulk of graphitic carbon nitride (*ACS Catal.* 2020, **10**, 11069, and *Nat. Nanotech.*, 2022, **17**, 174-181.), which might explain the relative high value.

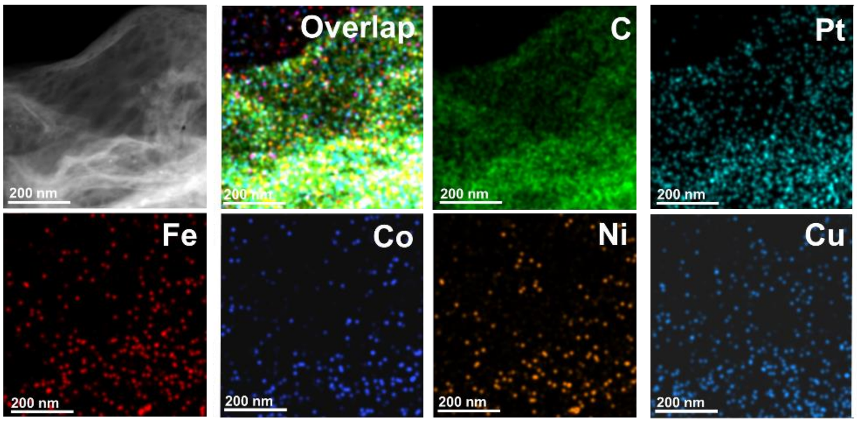

Supplementary Figure 37. EDS element mapping of PtFeCoNiCu HESACs. All metals are distributed uniformly in the HESACs.

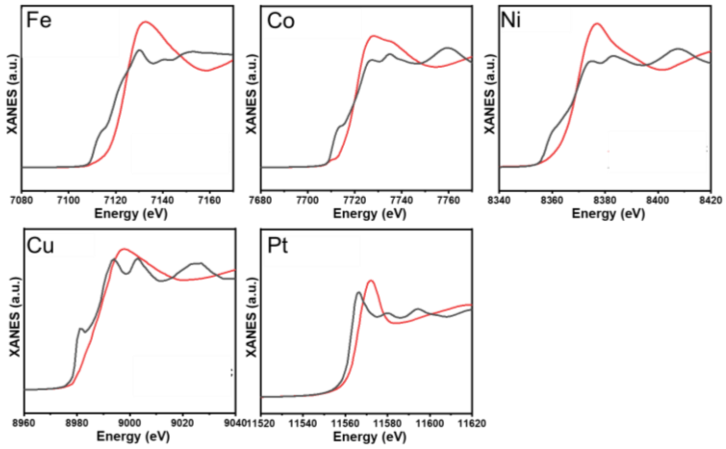

Supplementary Figure 38. XANEs spectra of metal in PtFeCoNiCu HESACs. Red line represents the as prepared catalyst and black line represents metal foil. All metals in HESACs show positive oxidation state based on the comparison on the intensity of the white line.

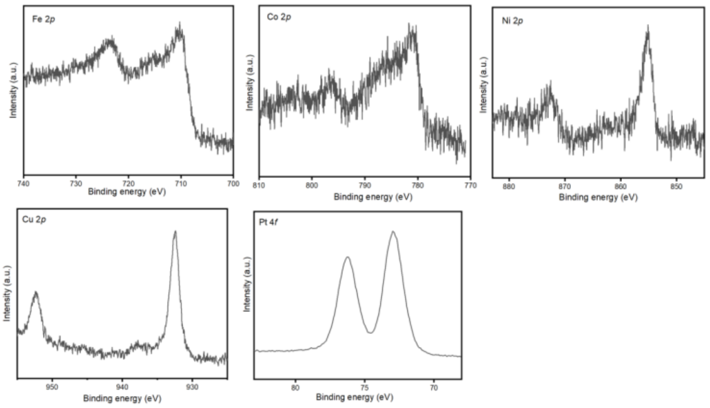

Supplementary Figure 39. XPS spectra of the metal in PtFeCoNiCu HESACs. The XPS results are in good agreement with the XANES spectra, further confirm the positive oxidation state of the metals in HESACs.

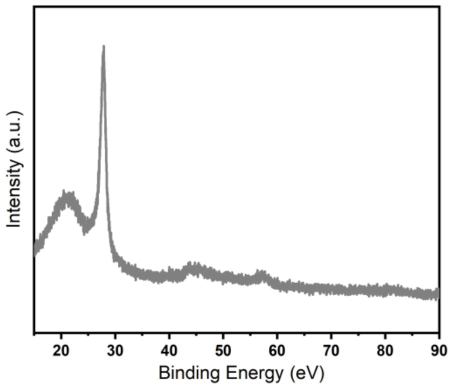

Supplementary Figure 40. XRD pattern of PtFeCoNiCu HESACs. No crystalline metal is detected.

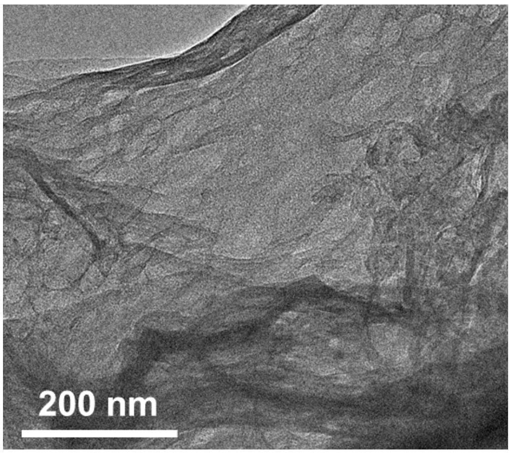

Supplementary Figure 41. TEM image of PtFeCoNiCu HESACs. No metal particles are observed.

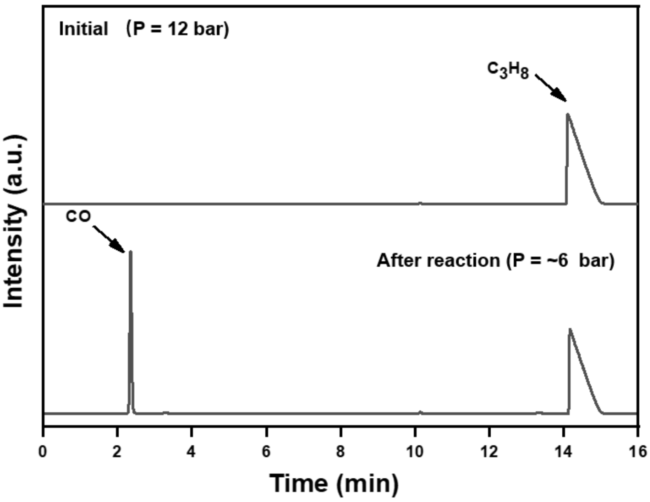

Supplementary Figure 42. GC analysis of the gas phase before and after the reaction. The initial pressure includes 1 atm air in reactor. The decreasing of the reaction pressure suggests the consumption of propane.

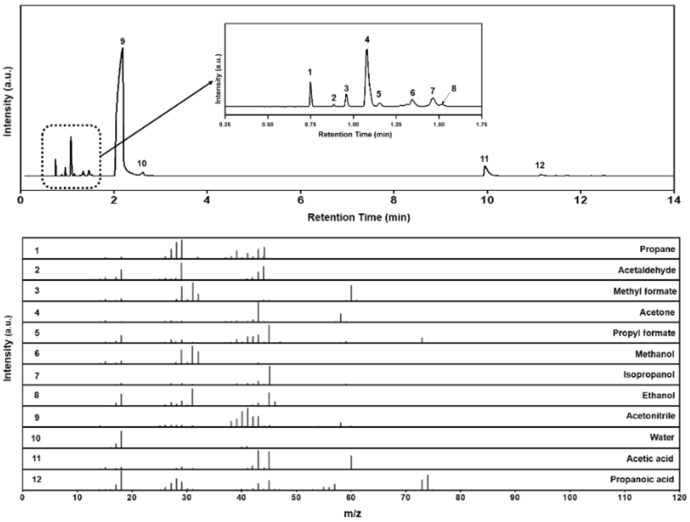

Supplementary Figure 43. GC-MS product profiles of the liquid phase and the mass spectra of various peaks.

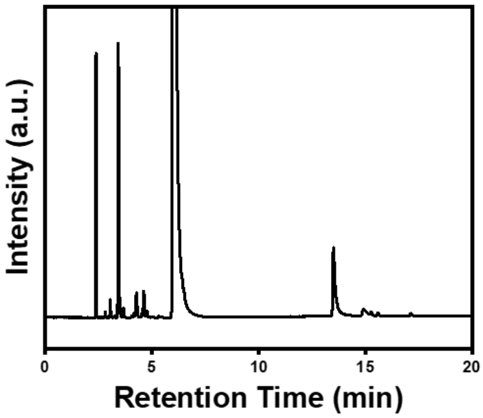

Supplementary Figure 44. GC-FID product profiles of the liquid phase. The peaks are identified by GC-MS measurement.

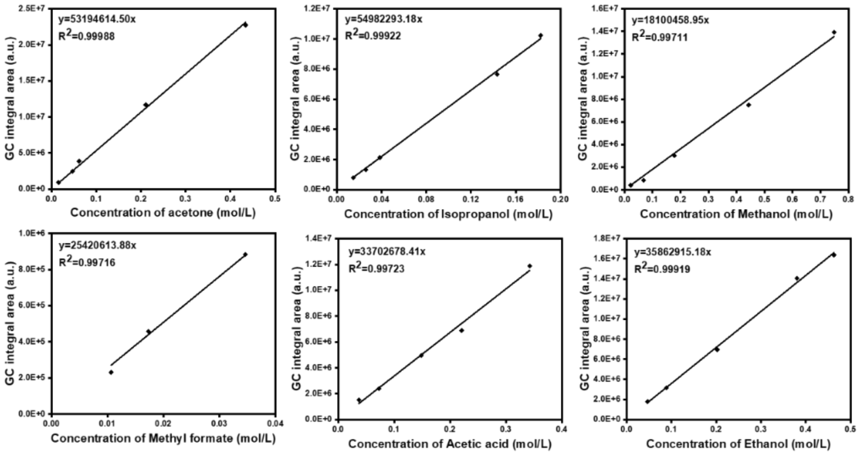

Supplementary Figure 45. External standard curves of various oxy-compounds based on the GC-FID results.

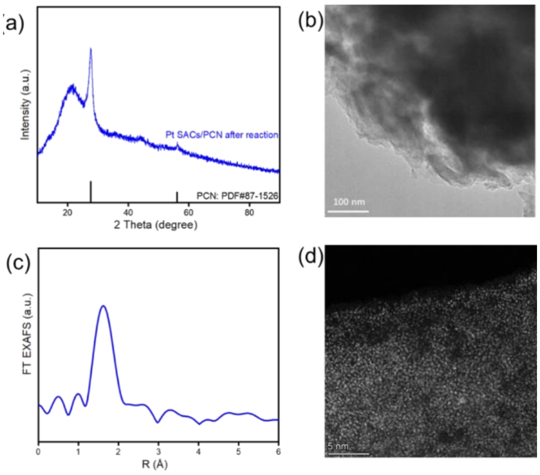

Supplementary Figure 46. Characterizations of Pt SACs/PCN after catalytic reaction. (a) XRD pattern. (b) TEM image. (c) Pt *L*-edge FT EXAFS spectrum. (d) Aberration-corrected HAADF-TEM image. The used catalyst maintains the dense isolated Pt sites.

Supplementary Table 1. Pt areal loading of reported Pt SACs.

	Sample	Pt areal density (atoms/nm^2)	Ref.
1	Pt_1/PCN	4.1	Nat. Nanotech., 2022, **17**, 174-181
2	Pt_1/CeO_2	3.9	Angew. Chem. Int. Ed., 2022, **61**, e202212338
3	Pt/CeO_2	<1.2	Catal. Today 2024, **425**, 114298
4	Pt_1/Fe_2O_3	1.2	Nanotechnology, 2018, **29**, 204002
5	$Pt(0.25)/TiO_2$	0.1	Nature Commun., 2024, **15**, 998
6	Pt_1-N/BP	0.01	Nature Communications, 2017, 8, 15938
7	$Pt_{1.1}/BP_{defect}$	0.02	Angewandte Chemie, 2018, 131, 4, 1175
8	S-Pt-C_3N_4	<0.5	Angewandte Chemie, 2020, 132, 15, 6283
9	Pt_1-CN	<0.5	Advanced Materials, 2016, 28, 12, 2427
10	Pt_1/Fe_2O_3	<0.5	Nature Communications, 2019, 10, 4500
11	Pt_1/Al_2O_3	0.9	Science Advances, 2020, 6, 25
12	Pt_1/NC	<0.5	Nature Communications, 2019, 10, 1278
13	PtSA-MNSs	0.6	Angewandte Chemie, 2019, 58, 30, 10198
14	20Pt/meso S-C	0.9	Science Advances, 2019, 5, 10
15	Pt SACs	<0.5	Nature Communications, 2019, 10, 4585

Supplementary Table 2. Metal content measured by ICP.

Sample	Metal	Content (wt%)	
Pt SACs/PCN	Pt	41.8	
Pt NPs/PCN	Pt	40.9	
V SACs/PCN	V	28.4	
Cr SACs/PCN	Cr	32.2	
Mn SACs /PCN	Mn	31.6	
Fe SACs /PCN	Fe	34.6	
Co SACs /PCN	Co	41.3	
Ni SACs /PCN	Ni	37.1	
Cu SACs /PCN	Cu	44.8	
Zn SACs /PCN	Zn	39.4	
Nb SACs /PCN	Nb	33.9	
Mo SACs /PCN	Mo	27.3	
Ir SACs /PCN	Ir	38.7	
Au SACs /PCN	Au	30.0	
Pt SACs /NC	Pt	34.1	
Fe SACs /NC	Fe	21.5	
Co SACs /NC	Co	19.6	
Ni SACs /NC	Ni	17.7	
Cu SACs /NC	Cu	29.8	
HESACs	Pt	15.6	Total: 32.4
	Fe	3.1	
	Co	4.1	
	Ni	2.3	
	Cu	7.3	

Supplementary Table 3. EDS element analysis measured with the operando aberration-corrected HAADF-TEM.

Annealing condition	Temperature (°C)	Atomic fraction (%)		Pt/Cl
		Pt	Cl	
In Vacuum	20	55.4	44.6	1.24
	200	90.9	9.1	9.99
	300	92.7	7.3	12.70
	400	95.8	4.2	22.81
In Ar	20	54.7	45.3	1.21
	200	82.8	17.2	4.81
	300	90.1	9.9	9.10
	400	95.3	4.7	20.28

Supplementary Table 4. Comparison on the catalytic performance of propane oxidation with molecule oxygen and other oxidants.

	Catalyst	Oxidant	Production	Temp. (°C)	Mass activity ($mmol/g_{cat}/h$)	TOF ($mol_{pro}·mol_M^{-1}·s^{-1}$)	Ref.
1	41.8 % Pt SAC/PCN	O_2	oxygenates	175	12.0	1.6×10^{-3}	This work
2	17 % Pt SAC/PCN	O_2	oxygenates	175	6.7	2.1×10^{-3}	This work
3	34.1 % Pt SAC/NC	O_2	oxygenates	175	6.1	0.96×10^{-3}	This work
4	Cu powder dispersed in 1.0 M $HClO_4$	O_2	propylene	25	1.8	3.3×10^{-5}	*Nat. Catal.*, 2023, 6, 666-675
5	TiO_2	O_2	CO_2	UV	1.1	2.4×10^{-5}	*J. Catal.*, 2015, **324**, 119-126
6	$LaCo_{0.1}Mn_{0.9}O_3$	O_2	CO_2	260	1.0	6.3×10^{-5}	*J. Phys. Chem. C*, 2020, **124**, 14646-14657
7	Immobilised iron complex	H_2O_2	oxygenates	50	-	2.8×10^{-3}	*Catal. Sci. Technol.*, 2023, **13**, 4839-4846
8	Tricopper cluster complex	H_2O_2	oxygenates	25	11.3	7.1×10^{-2}	ACS Sustainable Chem. Eng., 2018, **6**, 5431-5440
9	$CoCl_{16}Pc$-Na-X (0.27)	TBHP and O_2	oxygenates	25	-	1.7×10^{-2}	*Catal. Today*, 1999, **49**, 171-175
10	$CuCl_{16}Pc$-Na-Y (0.11)		oxygenates	25	-	1.3×10^{-2}	
11	$Tl(TFA)_3$ homogeneous	$Tl(TFA)_3$	oxygenates	180	1.6	8.1×10^{-5}	*Science*, 2014, **343**, 1232-1237
